# Current Hospital Topics

**Published:** 1907-02-09

**Authors:** 


### HOSPITAL ADMINISTRATION.

### CONSTRUCTION AND ECONOMICS.

### CURRENT HOSPITAL TOPICS.

#### Hospital Railway Cars.

American railway companies are introducing into their system the use of cars fitted up with all necessary surgical appliances so as to assume the nature of a hospital on wheels. These cars are intended for use in cases of accident at a distance from a hospital. The immense distances traversed by some of the lines through sparsely populated districts render such a provision for accident emergencies the more necessary. The Erie Railway Company has constructed a hospital car which we understand has been pronounced by surgeons to be equipped in the most complete and perfect manner possible. It is 60 feet long and divided into two compartments, one of which is fitted up as an operating-room with table, sterilisers, and all other necessary appliances. The other compartment is arranged as a ward-room with 11 bedsteads and a lavatory and saloon. Both parts are provided with sliding doors, so that the patients can be conveyed to the car and from one division to the other in stretchers. Hot and cold water is provided from tanks just under the roof of the car. Six-wheel bogeys are used so as to insure the utmost freedom from vibration. A similar hospital car has also been adopted by the Southern Pacific Railway Company at a cost of £3,600. This car is 76 feet long, and runs upon two six-wheel bogeys. Under ordinary circumstances this car can be used partly as a drawing-room and the rest as a sleeper, but the whole can be rapidly transformed into a " hospital " with operating-room and ward similar to the car described above.

#### The Hospital Saturday Fund.

Mr. Reginald B. D. Acland, K.C., at the annual dinner of the Hospital Saturday Fund presented Mr. W. G. Bunn with a beautifully illuminated address and a cheque for £134 on his retirement from the post of secretary. Mr. Acland affirmed that the Hospital Saturday Fund had now a real position—very different from what it had been in the old days—among the charitable agencies of London, a position won by a great deal of hard work. Mr. Acland dwelt upon the difficulties of the old days, and reminded his audience that although the first year's collections exceeded £5,000, at the end of another thirteen years the total sum raised had only been increased by £733. At the present day, in lieu of the dark offices in a court leading out of Fleet Street, the Saturday Fund had its own freehold premises, which were fitted up to enable them to do their work in comfort, and they had a proper staff, sound organisation and the good wishes of everybody. Mr. Acland did not claim that all this progress was due to Mr. Bunn, but he said, without fear of contradiction, that the Fund never could have made the progress it had without a man like Mr. Bunn, who combined within himself many qualities. Mr. Bunn possessed a perfect knowledge of what were called the working classes, the confidence of the great friendly societies of which he was so distinguished an ornament, absolute integrity, unfailing courtesy, a great power of speech, and an unwearying power of work. The Fund now raised £26,000 a year, and Mr. Acland looked forward to the time when it would distribute at least £50,000 per annum amongst the hospitals and medical charities.

#### Hospitals and Taxation.

For more than forty years efforts have been made over and over again to exempt hospitals from rates. A century and a half ago the hospitals paid neither rates nor taxes, as public opinion in those days held that gifts for the sick poor were in reality gifts to God which should be free from all imposts of the kind. Many things have happened in the interval which have necessitated a reconsideration of this position, and have led to the taxation of all public institutions, whether devoted to charity or otherwise. Nearly forty years ago the agitation to exempt hospitals from rating took a very practical form, and under the ægis of a society for the exemption of hospitals from taxation much work was done, which resulted in large majorities being obtained in the House of Commons in favour of the principle of non-taxation. The matter was then taken up by Mr. Gladstone's Government, and after conference with the Opposition, it was determined to submit Government buildings to taxation in order to affirm the principle that every building in a great city, occupying an important site, should be liable to be rated. An illustration of the desirability of this policy was afforded by the history of the city of Quebec, where the principal sites were occupied by institutions, chiefly religious, which paid no rates or taxes. This position largely increased the rates which had to be levied on ordinary people in that city, and tended for many years to cripple the city's resources, and to prevent developments which were of great importance to the well-being of the inhabitants as a whole. A movement has been started in England, with the object of abolishing the legacy duty paid by hospitals. In our view, however, a question of this kind cannot be dealt with piecemeal. It is necessary first of all to determine the principle of taxation and rating to be applied to all properties, and in no other way is it practically possible to hope that any remission will be obtained in favour of hospitals.

### Strength in Little Things.

During the last ten days we have received probably one dozen appeals, sometimes three in a day by different posts, from the same source. The sender is a lady who lives in Worcestershire, and who asks for contributions to her Lenten collection. Each envelope contains six documents, of the commonest kind and very indifferently printed. Two of them are cards, one for a shilling fund, and the other divided into spaces for a collection in pence, threepences, sixpences and shillings. The inter-spaces are quite erratic, and are based on no system for raising a definite sum under each heading. There is a four-page green bill, $10\frac{1}{2}$ inches long, containing a statement of accounts for the year 1906, duly audited, which is a curiosity in its way; a white bill, printed on both sides, 14 inches long, containing a list of people in reduced circumstances; a small red bill, printed on one side, urging that each card should bring in 6d., being one penny for each week in Lent; and a notice on a little white slip begging those who receive more than one appeal not to be annoyed by that fact. At the first glance it is impossible to imagine that anything more hopeless could be devised by way of appeal literature. Yet the accounts show that this literature last year produced upwards of £4,000; and that the total expenditure on postage, printing, clerical expenses and the auditor amounted to a little over £500, or say thirteen per cent. Who can maintain that appeals through the post are unproductive in the present day, when judged by the instance we have just given? It is further an undoubted fact that missionary societies raise several millions every year by similar methods.

